# Antigen-specific assessment of the immunological status of various groups in a leprosy endemic region

**DOI:** 10.1186/s12879-015-0962-4

**Published:** 2015-05-30

**Authors:** Angélica da Conceição Oliveira Coelho Fabri, Ana Paula Mendes Carvalho, Sergio Araujo, Luiz Ricardo Goulart, Ana Márcia Menezes de Mattos, Henrique Couto Teixeira, Isabela Maria Bernardes Goulart, Malcolm S. Duthie, Rodrigo Correa-Oliveira, Francisco Carlos Félix Lana

**Affiliations:** Department of Basic Nursing, Faculdade de Enfermagem, Universidade Federal de Juiz de Fora - UFJF, Juiz de Fora, MG Brazil; Postgraduate Program in Nursing, Escola de Enfermagem, Universidade Federal de Minas Gerais - UFMG, Belo Horizonte, MG Brazil; National Reference Center for Sanitary Dermatology and Leprosy - CREDESH, Hospital das Clínicas, Universidade Federal de Uberlandia - UFU, Uberlândia, MG Brazil; Institute of Genetics and Biochemistry, Universidade Federal de Uberlandia - UFU, Uberlândia, MG Brazil; Department of Medical Microbiology and Immunology, University of California-Davis, Davis, CA USA; Postgraduate Program in Biological Science - Immunology and Infectious Parasitic Disease, Instituto de Ciências Biológicas, Universidade Federal de Juiz de Fora - UFJF, Juiz de Fora, MG Brazil; Department of Parasitology, Microbiology and Immunology, Instituto de Ciências Biológicas, Universidade Federal de Juiz de Fora - UFJF, Juiz de Fora, MG Brazil; Infectious Disease Research Institute, Seattle, WA USA; Laboratory of Cellular and Molecular Immunology, Centro de Pesquisas René Rachou - CPqRR, Fundação Oswaldo Cruz - FIOCRUZ, Belo Horizonte, MG Brazil; Laboratory of Immunology, Núcleo de Pesquisas em Ciências Biológicas, Universidade Federal de Ouro Preto - UFOP, Ouro Preto, MG Brazil; Instituto Nacional de Ciência e Tecnologia em Doenças Tropicais - INCT-DT, Belo Horizonte, Brazil; Department of Maternal and Child Nursing and Public Health, Escola de Enfermagem, Universidade Federal de Minas Gerais - UFMG, Belo Horizonte, MG Brazil

## Abstract

**Background:**

Serological tests can be important tools to assist in the diagnosis of leprosy and can contribute to an earlier diagnosis. The aim of this study was to evaluate the antibody responses against phenolic glycolipid-1 (PGL-1), natural disaccharide linked to human serum albumin via an octyl (NDO-HSA), Leprosy IDRI Diagnostic-1 (LID-1) and natural disaccharide octyl - Leprosy IDRI Diagnostic-1 (NDO-LID) in leprosy patients, household contacts of patients and the general population.

**Methods:**

Enzyme-linked immunosorbent assays were used to analyze the antigen-specific antibody responsesof 94 leprosy cases, 104 household contacts of cases and 2.494 individuals from the general population.

**Results:**

A positive correlation was observed for the antibody responses to all antigens studied. A higher proportion of seropositivity for all antigens, along with stronger magnitude of response, was observed in multibacillary (MB) leprosy patients and household contacts of MB leprosy patients compared with the levels observed in paucibacillary (PB) leprosy patients and household contacts of PB leprosy patients. A substantial and significant positive correlation was found between seropositivity and the bacterial index for the leprosy patients. Anti-PGL-1 tests were more frequently positive than anti-NDO-HSA tests among patients with all clinical forms of leprosy and among the group of household contacts. The LID-1 and NDO-LID antigens showed a greater capacity to identify household contacts and individuals from the general population infected with *M. leprae.*

**Conclusions:**

Tests that measure the antibody responses against LID-1, NDO-LID, NDO-HSA and PGL-1 were effective tools for the detection of patients with MB leprosy. Our data indicate that the anti-LID-1 and anti-NDO-LID responses were more effective than an anti-NDO-HSA response for the identification of individuals with subclinical infection.

## Background

Leprosy presents across a wide range of symptoms defined into 5–6 categories within the Ridley-Jopling scale, although for treatment purposes these are simplified/ consolidated to either multi- or paucibacillary (MB and PB, respectively). The diagnosis of leprosy is not simple and, not surprisingly, many professionals have neither the experience to recognize the various signs and symptoms of the disease nor the ability to differentiate them from other diseases [1]. Thus, leprosy patients often receive incorrect diagnoses and appropriate treatment is delayed.

Antibody responses to specific *M. leprae* antigens can be evaluated by several tests. Among these are serologic tests that measure the levels of immunoglobulin M (IgM) against phenolic glycolipid-1 (PGL-1) (which may be detected by either native (anti-PGL-1) [2] or the synthetic mimetope natural disaccharide, typically linked to human serum albumin via an octyl (NDO-HSA) [3]) IgG against leprosy IDRI diagnostic 1 (LID-1) [4, 5] or both IgM and IgG against natural disaccharide octyl - leprosy IDRI diagnostic 1 (NDO-LID) [1]. The titers of antibodies against PGL-1, LID-1 and NDO-LID vary with clinical presentation, being highest in the lepromatous - lepromatous (LL) clinical form and lowest, or absent, in the tuberculoid - tuberculoid (TT) form. The antibody titers generally increase as the disease progresses across the spectrum from the TT to the LL form. The bacterial index (BI) similarly varies and is positively correlated with antibody titers [1, 5, 6].

Individuals living in leprosy endemic areas, which are typically impoverished and have high population densities, are commonly infected with *M. leprae* [7]. Thus, the possibility that asymptomatically infected individuals may be involved in the *M. leprae* transmission chain should not be overlooked [8]. Subclinical *M. leprae* infection in endemic populations is traditionally assessed using either enzyme-linked immunosorbent assays (ELISA) or lateral flow point-of-care (POC) tests to detect specific antibodies [8, 9]. When used in this way these assays may enable earlier identification and treatment of patients, and thus contribute to both the prevention of physical disabilities and the reduced transmission of *M. leprae* [4].

It has been demonstrated in various settings that household contacts of untreated MB patients experience greater exposure to *M. leprae* than the general population (GPop) [6]. Anti-PGL-1 seropositivity in household contacts of leprosy patients has been associated with an increased risk of developing disease [9]. It is important, however, to consider that individuals living in highly endemic regions may be routinely exposed to *M. leprae* even if they do not live with a recognized patient. It therefore becomes pertinent to understand the performance of tests involving the PGL-1, LID-1, NDO-LID and NDO-HSA antigens in different contexts and groups within the population. The aim of this study was to evaluate serum antibody responses against PGL-1, NDO-HSA, LID-1 and NDO-LID in patients diagnosed with leprosy, in household contacts of leprosy patients and among the general population of a leprosy endemic region municipalities with varying leprosy detection rates.

## Methods

### Patient and contact samples

Untreated patients (n = 94) and household contacts (n = 104) were recruited at the National Reference Centre for Sanitary Dermatology and Leprosy (CREDESH), Uberlandia, Minas Gerais, Brazil, a public health care facility in an endemic region where routine prevention, including Bacillus Calmette–Guérin (BCG) vaccination, household contact monitoring, early case detection, and treatment are available and under constant supervision. The Uberlândia municipality had detection rate of 10.81/100.000 inhabitants in 2012 [10].

Leprosy patients were diagnosed after thorough dermato-neurological and laboratory examinations, and classified according Ridley-Jopling five-group system of clinical manifestations into: tuberculoid (TT), borderline tuberculoid (BT), mid-borderline (BB), borderline lepromatous (BL) or lepromatous (LL) [11]. For treatment purposes patients were also stratified into paucibacillary (PB), with up to five skin lesions and a negative bacilloscopy, or MB, with more than five lesions and/or positive bacilloscopy in accordance with the World Health Organization operational classification [12].

Household contacts (HHC) who resided with leprosy patients, or had resided with leprosy patients in the five years prior to diagnosis, were examined for signs or symptoms that were suggestive of leprosy by physicians with specialized leprosy training. Most HHC were relatives of their index case (spouse, parent or sibling). HHC were stratified according to the operational and clinical classifications of their index case.

### Samples from the general population

Individuals from the general population (GPop; n = 2.494) were selected randomly from seven municipalities in the microregion of Almenara, Minas Gerais, Brazil [13], which had a mean detection rate of 31.32/100.000 inhabitants in 2012 [10]. Finger-prick blood spots were collected on Whatman 3 MM paper (Whatman, Maidstone, UK) and stored at 4 °C until serum was eluted by adding 1 % bovine serum albumin in 1X phosphate buffered saline. Based on estimates of the volume of whole blood in a 2.5 mm filter paper disc, the final dilution of the eluted serum was 1:100 [14, 15].

### Antibody detection

Antigen-specific IgM and IgG antibodies were measured by indirect enzyme-linked immunosorbent assays (ELISA) as previously described [3]. Briefly, the four antigens (PGL-1, NDO-HSA, LID-1 and NDO-LID) were used to coat 96-well microtiter plates (LABWARE Manufacture CO, People’s Republic of China); 1 μg/mL of LID-1 or 0.2 μg/mL of NDO-HSA, NDO-LID or PGL-1 was added per well in 100 μL of 0.1 M sodium carbonate/bicarbonate buffer, pH 9.6, and incubated at 4 °C overnight. The assay was performed using serum samples at a dilution of 1:300 and whole blood samples at a dilution of 1:100 [14, 15]. After blocking for 1 h at 37 °C, detection antibodies were similarly incubated for 1 h at 37 °C, after which four washes were performed. The wells were then treated with ortho-phenylenediamine (OPD) substrate, and the absorbance at 492 nm was obtained using a spectrophotometer plate reader (Molecular Devices, Sunnyvale, CA). To minimize inter- and intra-test errors, an ELISA index (EI) was calculated as follows: the EI equals the optical density (OD) of the sample divided by the OD of the cut-off [5]. The cut-off was calculated as an average of three controls negative (individuals living in low endemic areas leprosy and which showed result of the ML Flow test negative) plus three times the standard deviation, such that samples with EI values of 1.1 or above were considered to be positive.

### Statistical analysis

Graphs and mean values were generated using GraphPad Prism version 5 (GraphPad Software Inc., La Jolla, CA, USA), and statistical analysis was performed using Statistical Package for the Social Sciences (SPSS) version 18 (SPSS Inc., Chicago, IL, USA). Statistical significance was assessed using nonparametric methods, with the Kruskal-Wallis one-way (H) analysis of variance used to make comparisons among multiple groups and the Mann–Whitney *U* test with Bonferroni correction used to make comparisons between two groups. Spearman’s coefficient (rho) was used to test the strengths of correlations. Results were considered statistically significant when *p*-values ≤0.05 were obtained or when *p*-values ≤0.0167 using the Mann–Whitney *U* test. The concordance among the four antigens was calculated using the Kappa coefficient. The NDO-HSA antigen was selected as the reference antigen because it was tested in three groups (patients, HHC and GPop) and because it is one of the most common antigens used in tests for *M. leprae*. Kappa values and their interpretation varied as follows: <0, no agreement; 0–0.20, poor agreement; 0.21-0.40, fair agreement; 0.41-0.60, moderate agreement; 0.61-0.80, substantial agreement; and 0.81-1.00, almost perfect agreement [16].

### Ethics statement

This study conforms to the Declaration of Helsinki and was reviewed and approved by Research Ethic Committee (CEP) of the Federal University of Uberlandia, Protocol Number CEP/UFU 138/08, and the Research Ethic Committee (COEP) of the Federal University of Minas Gerais, Protocol Number ETIC 158/09. All participants signed an informed consent form and authorized the collection of the samples. The informed consent form for children under 18 years of age was signed by either a parent or a legal guardian.

## Results

### Antibody responses among leprosy patients

**Fig. 1:**
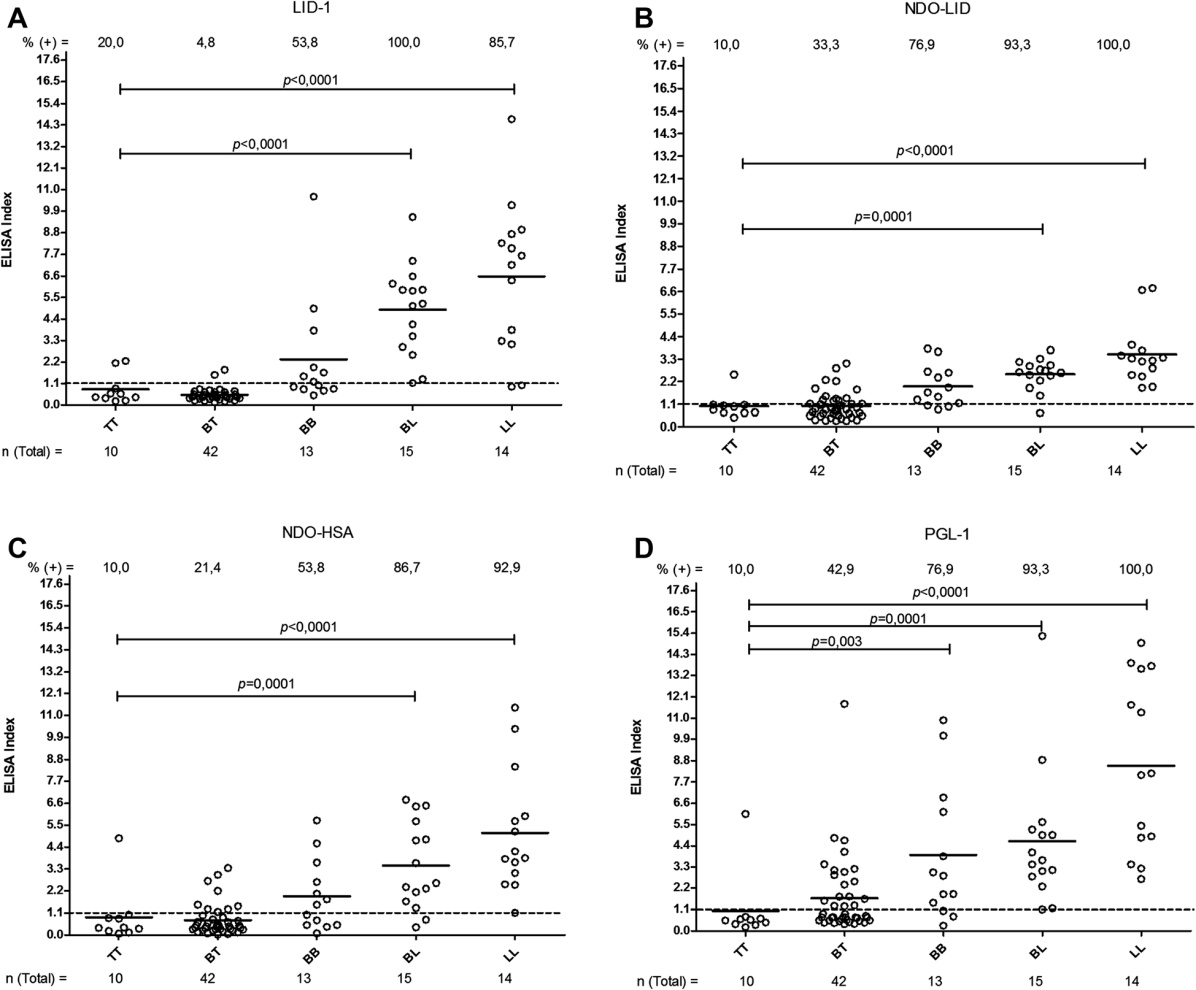
Antigen-specific responses of leprosy patients, stratified by antigen. Sera from fully characterized leprosy patients were analyzed for antibodies against **a**. LID-1 (IgG); **b**. NDO-LID (IgG and IgM); **c**. NDO-HSA (IgM) and **d**. PGL-I (IgM). Each point represents the EI of an individual serum sample. The mean EI is represented by the horizontal line. The traced horizontal line is threshold for determining a positive result (EI = 1.1). The number above each data set is the percentage of positive results, and the number below each data set represents the total number of participants in each group

**Fig. 2:**
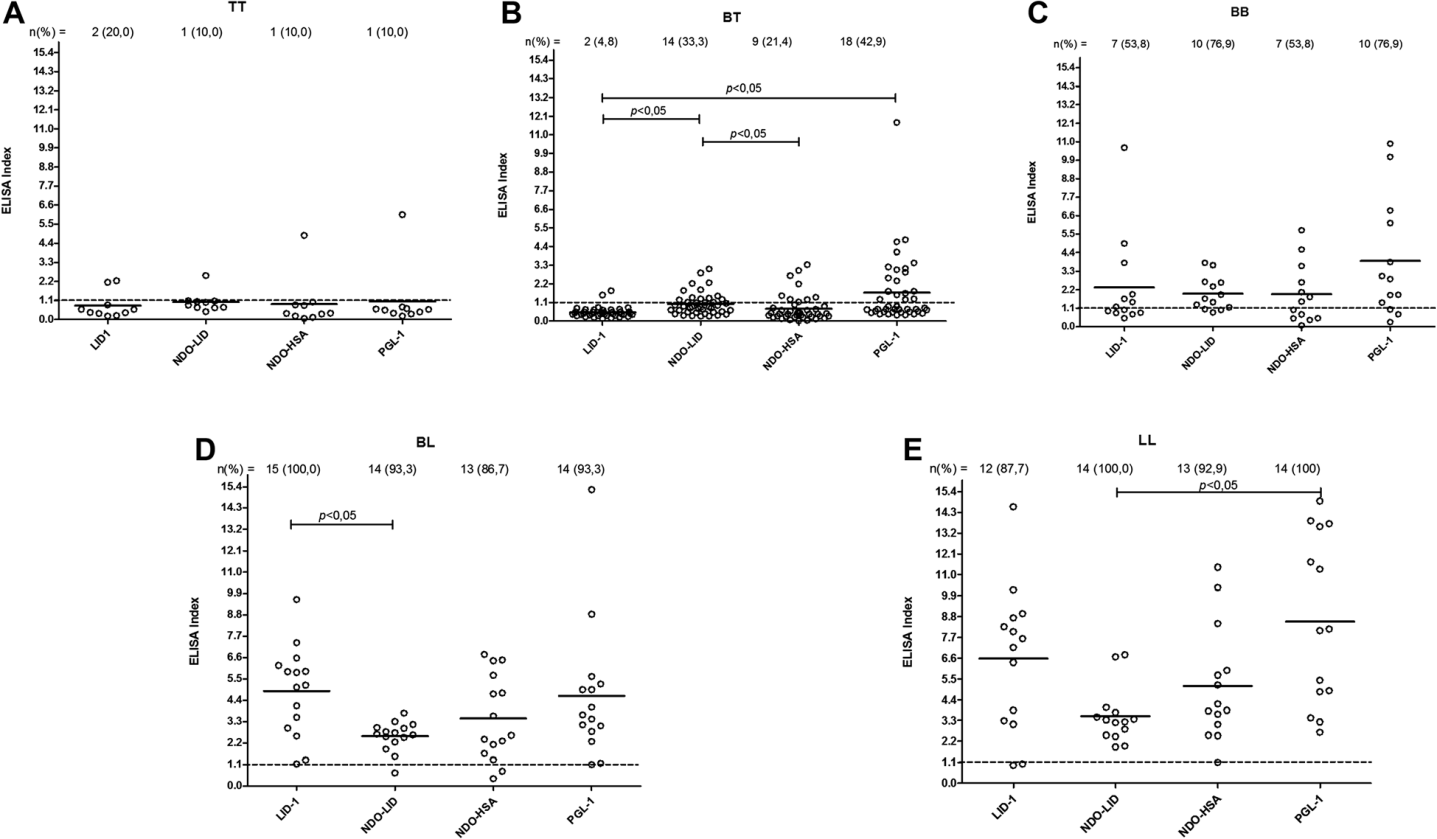
Antigen-specific responses of leprosy patients, stratified by Ridley-Jopling classification. Sera from leprosy patients characterized as **a**. TT; **b**. BT; **c**. BB; **d**. BL or **e**. LL were analyzed for antibodies against LID-1 (IgG), NDO-LID (IgG and IgM), NDO-HSA and PGL-I (IgM). Each point represents the EI of an individual serum sample. The mean EI is represented by the horizontal line. The traced horizontal line is threshold for determining a positive result (EI = 1.1)

We determined the presence of antigen-specific antibodies in the serum samples from various groups. In the group of patients, as anticipated the seropositivity of antibodies against LID-1, NDO-LID, NDO-HSA and PGL-1 were highest in MB patients and lowest in PB patients (BT and TT forms). Antibodies against NDO-LID (28.3 %) and PGL-1 (33.3 %) were, however, better able to detect PB patients than both LID-1 (10.5 %) and NDO-HSA (23.3 %) (Table 1). Further analyses within the context of the Ridley-Jopling scale demonstrated that 100 % (n = 14) LL patients were seropositive for anti-NDO-LID and anti-PGL-1, while 85.7 % (n = 12) and 92.9 % (n = 13) were seropositive for anti-LID-1 and anti-NDO-HSA, respectively. Notably, the seropositivity rates for antibodies against the 4 antigens evauated were significantly different between the TT and LL groups (Fig. 1). The NDO-LID antigen had a higher seropositivity rate than LID-1 and NDO-HSA in the BT group. In the BL group the LID-1 antigen had a higher seropositivity rate than NDO-LID, and in LL group PGL-1 was observed to have a higher seropositivity than NDO-LID (Fig. 2). In addition, a positive correlation was observed for the bacterial indices (BI) and the antibody titers against LID-1 (rho = 0.81), NDO-LID (rho = 0.67), NDO-HSA (rho = 0.60) and PGL-1 (rho = 0.61) (all *p* < 0.0001). Thus, our data indicate that EI increase with BI and across the disease spectrum.

**Table 1:** Proportion of seropositivity for antibodies against LID-1, NDO-LID, NDO-HSA and PGL-1 according to groups of individuals

			No. (%) positive^a^			
		No. of samples	LID-1	NDO-LID	NDO-HSA	PGL-1
Patients	MB	43	34(89.5)	38(71.7)	33(76.7)	38(66.7)
	PB	51	4(10.5)	15(28.3)	10(23.3)	19(33.3)
HHC	MB^b^	81	35(43.2)	31(38.3)	10(12.3)	14(17.3)
	PB^c^	23	6(26.1)	6(26.1)	0(0.0)	1(4.3)
GPop		2494	74(21.9)	995(41.2)	191(7.9)	-

^a^Test were considered positive when a distinct band was observed (scored as EI of 1.1 or greater)

^b^HHC of patients classified as MB

^c^HHC of patients classified as PB

### Responses of household contacts (HHC) and the general population (GPop)

It is documented that HHC are at higher risk of *M. leprae* infection and development of disease than the general population. We therefore analyzed the antigen-specific antibody responses of HHC and a random selection of residents (GPop) within the leprosy endemic region of Minais Gerais. The seropositivity rate was higher among the HHC of MB patients than those of PB patients, supporting the hypothesis that HHC of MB patients are exposed and more likely to be infected with *M. leprae* (Table 1). Positive responses were also detected among the GPop. Together, these data suggest a large proportion may be harboring *M. leprae* without any clinical symptoms of disease.

### Relationship of responses

**Fig. 3:**
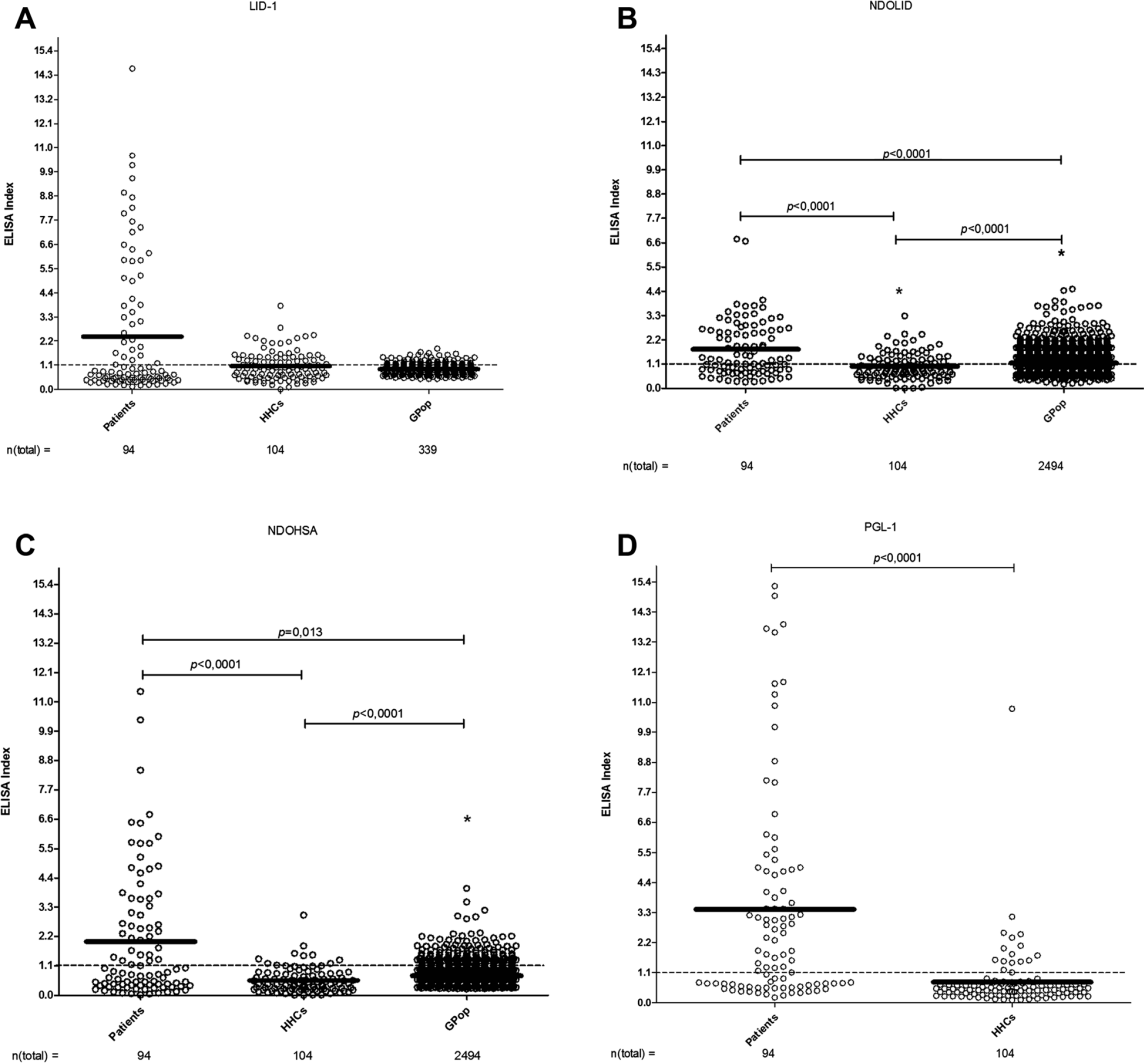
Antigen-specific responses among the broader population. Sera from multiple groups were analyzed for antibodies against **a**. LID-1 (IgG); **b**. NDO-LID (IgG and IgM); **c**. NDO-HSA (IgM) and **d**. PGL-I (IgM). Each point represents the EI of an individual serum sample, with the total number of participants in each group noted below. The mean EI is represented by the horizontal line. The traced horizontal line is threshold for determining a positive result (EI = 1.1). **a**: IgG against LID-1: Patients *versus* HHCs *versus* Gpop, difference not significant (*H* = 0.57; *p* = 0.753). **b**: IgG and IgM against NDOLID: Patients *versus* HHCs *versus* Gpop (H = 31.37). Mann–Whitney *U* test with Bonferroni correction: Patients *versus* HHCs (*p* < 0.0001); Patients *versus* GPop (*p* < 0.0001); HHCs *versus* GPop * mean EI of the GPop group was greater than the mean EI of the HHCs group (*p* < 0.0001). **c**: IgM against NDOHSA: Patients *versus* HHCs *versus* GPop (H = 50.66). Mann–Whitney *U* test with Bonferroni correction: Patients *versus* HHCs (*p* < 0.0001); Patients *versus* GPop (*p* = 0.013); HHCs *versus* GPop * mean EI of the EC group was greater than the mean EI of the HHCs group (*p* < 0.0001). **d**: IgM against PGL-1: Patients *versus* HHCs (*U* = 1769; *p* < 0.0001)

Significant differences were observed between the EI for antibodies to NDO-LID and NDO-HSA in the patient, HHC and GPop. It is noteworthy that for NDO-LID and NDO-HSA the mean EI of GPop was higher than that of HHC. A significant difference was also observed between the patient group and HHC for PGL-1 (Fig. 3).

The concordance between the anti-NDO-HSA and anti-LID-1 (k = 0.63), anti-PGL-1 (k = 0.62) and anti-NDO-LID (k = 0.79) was substantial and significant in the patient group (all *p* < 0.0001). In GPop the agreement was poor, while in HHC fair agreement was observed between the result of antibodies against NDO-HSA and NDO-LID (k = 0.32; *p* < 0.0001).

Using the Spearman test, a significant positive correlation was identified between the different antigens tested in GPop as well as in the patients. In HHC, no significant correlation was identified for any combination with the PGL-1 antigen (Table 2). We observed that the correlation was higher in the group of patients.

**Table 2:** Results of the *Spearman* test in different groups of individuals according to antigen

	GPop		HHC		Patients	
Antigens	rho	p-value	rho	p-value	rho	p-value
LID-1 and NDO-LID	0.635	<0.0001	0.345	0.0003	0.816	<0.0001
LID-1 and NDO-HSA	0.506	<0.0001	0.434	<0.0001	0.726	<0.0001
NDO-LID and NDO-HSA	0.323	<0.0001	0.864	<0.0001	0.875	<0.0001
PGL-1 and NDO-LID	-	-	0.150	0.129	0.727	<0.0001
PGL-1 and NDO-HSA	-	-	0.134	0.097	0.718	<0.0001
LID-1 and PGL-1	-	-	−0.122	0.219	0.646	<0.0001

rho: Spearman coefficient

LID-1: Leprosy IDRI diagnostic 1

NDO-LID: Natural disaccharide octyl - leprosy IDRI diagnostic 1

NDO-HSA: Natural disaccharide linked to human serum albumin via an octyl

PGL-1: Phenolic glycolipid-1

## Discussion

We examined the presence of antibodies against particular *M. leprae* antigens among various groups living in a leprosy endemic region. Consistent with previous observations [1, 5, 6], positive correlations with patient BI were observed for all of the antigens. Moreover, higher seropositivity was observed for anti-LID-1, anti-NDO-LID, anti-NDO-HSA and anti-PGL-1 in MB patients (BL, LL and some BB patients) and in HHC of MB patients than in PB patients and their HHC. A large proportion of the general population were also found to be seropositive. Together, our data suggest that a relatively large proportion of the population may harbor *M. leprae* infection.

In agreement our findings, an earlier comparison of antibodies against either native PGL-1 or semisynthetic NDO-HSA demonstrated significant agreement between these assays [3]. Our evaluations expand this observation and identify significant agreement across patient groups for seropositivity against the LID-1, NDO-LID, NDO-HSA and PGL-1. In contrast, such agreements were not observed in HHC or within the general population. We speculate that agreement in the antigen-specific antibody responses is observed in MB patients because they present with a high bacterial load and a more developed and defined humoral response. Of note, antibody responses to *M. leprae* appear to develop heterogeneously even in the controlled setting of experimental infection of armadillos [17], and thus, antigen-specific responses may develop at differing rates and be variable in infected individuals who have not yet exhibited signs of disease.

Some of the assays described here, for example ELISA detecting antibodies against the NDO-LID conjugate, appear to be more sensitive than others in the general population. The positive correlation observed for antibodies to the LID-1, NDO-HSA and NDO-LID antigens in patients, HHC and the general population infer that although the tests do not show perfect agreement, the value of the EI of a particular individual has a unidirectional trend.

The NDO-LID conjugate has previously demonstrated a greater capacity to identify patients with MB leprosy than the either of the NDO-HSA and LID-1 antigens alone [1]. Although serological tests appear to have a limited ability to aid the diagnosis of PB patients, our data also identified that a greater number of PB patients were seropositive for antibodies against NDO-LID than against either NDO-HSA or LID-1 alone, as has been reported previously [1, 6, 18]. This was especially true for patients categorized into the BT group, an effect similarly observed in a study of rapid diagnostic tests (NDO-LID, Orange Life®) [6]. These findings suggest the possibility of using NDO-LID-based tests within leprosy control programs to identify patients early in the clinical spectrum of leprosy and to identify PB patients.

Our data indicate that tests detecting antibodies to PGL-I and/or LID-1 represent effective tools for the detection of MB patients. Among all clinical forms of leprosy and among HHC the use of native PGL-1 resulted in a higher positivity rate than the synthetic mimetic of PGL-1 (NDO, conjugated to the inert carrier protein HSA). This result is in agreement with the literature, as a study comparing ELISA involving PGL-1 or NDO-HSA with the ML Flow rapid test indicated that native PGL-1 ELISA resulted in greater sensitivity and accuracy than either the NDO-HSA ELISA or ML Flow tests [3]. In our comparison of leprosy patients a higher mean EI was observed for PGL-1 than NDO-LID and LID-1. However, the need to extract native PGL-1 from the organs of *M. leprae*-infected armadillos is limiting and the sustainable use of synthetic NDO is preferred [19]. Relative to the NDO-HSA antigen, the NDO-LID conjugate demonstrated a greater potential to identify infected HHC and individuals from the general population who were infected with *M. leprae*. These tools could also be used to aid clinicians lacking expertize in leprosy diagnosis in general health care services to identify individuals at high risk of developing leprosy.

There is an epidemiological need for applicable diagnostic tools to detect asymptomatic individuals infected with *M. leprae* and those patients with early manifestations of leprosy. The periodic evaluation of seroprevalence rates in leprosy endemic areas may also be a useful measure of *M. leprae* transmission [20]. We expected to observe a greater positivity among HHC than in the general population because HHC are thought to be regularly exposed to *M. leprae* up until the patient is undergoing treatment [21]. On the contrary, however, we observed a higher rate of anti-NDO-LID and anti-NDO-HSA positivity in the general population than HHC, although a limitation of the study design was that the general population was from a different region than the patients and HHC. The higher endemicity rate in the region from which the general population samples were collected probably contributed to the higher seroposivity rate and leads us to suggest that that population is regularly being exposed to *M. leprae* and suggests that the hidden prevalence of leprosy may be quite high [22].

Serological tests involving synthetic PGL-1 antigen are used in some regions to identify individuals, especially HHC, infected with *M. leprae* [8, 9]. Our findings support the use of tests evaluating the anti-NDO-LID response, especially among HHC and in the general population of areas of high endemicity for leprosy. The application of anti-NDO-LID tests in the general population could assist leprosy control programs by allowing simple identification of a larger number of individuals infected with *M. leprae* and consequently those that have high risk of developing leprosy. Of particular relevance for the practicalities of large scale surveillance programs, our analysis of the general population examined whole blood collected on filter paper. A previous study demonstrated that samples stored in filter paper allowed for the recovery of antibody levels similar to those in serum samples [14] and that there is a close correlation between studies using venipuncture samples captured on filter paper [23, 24]. It is believed, therefore, that analysis of samples collected on filter paper was not a detrimental or limiting factor for our study.

The high positivity in the general population for antibodies to all of the studied antigens should raise concern because multiple reports suggest that individuals with subclinical *M. leprae* infection may be a potential transmission source for the infection of additional people [8]. As suggested by others, we support the georeferencing of patients and *M. leprae*-infected individuals to identify population clusters that might benefit from greater leprosy-specific vigilance by general health services. Furthermore, the quantitative measurement of antibodies against these antigens would appear beneficial in the selection of seropositive individuals for clinical examination and the earlier detection of leprosy cases.

## Conclusions

We observed high rates of seropositivity for antibodies against LID-1, NDO-LID, NDO-HSA and PGL-1 antigens among various groups within the population. High rates were observed for all antigens in MB patients and HHC of MB patients, and a positive correlation was observed between serology and BI; marked, significant agreement was found between these measures in leprosy patients, in particular, but a positive correlation was also observed in the general population.

The LID-1 and NDO-LID antigens showed a greater capacity to identify HHC and individuals of the general population infected with *M. leprae*. PGL-1 resulted in a higher degree of positivity than NDO-HSA for all clinical forms of leprosy and for HHC.

We suggest that NDO-LID represents an important antigen for the surveillance of HHC and of the general population. Our data provide evidence that all of the antigens tested are relevant tools to support the operational classification of leprosy and can also be used to identify individuals infected with *M. leprae*.

The results of this study indicate the need for greater vigilance in the health services in regions with high leprosy endemicity because many cases may currently be undiagnosed. These undiagnosed cases likely contribute to the continued transmission of *M. leprae* and maintenance of leprosy as an ongoing health concern.

## Abbreviations

POC: Point-of-care
PGL-1: Phenolic glycolipid-1
NDO-HSA: Natural disaccharide linked to human serum albumin via an octyl
IgG: Immunoglobulin G
LID-1: Leprosy IDRI Diagnostic-1
IgM: Immunoglobulin M
NDO-LID: Natural disaccharide octyl - leprosy IDRI diagnostic 1
LL: Lepromatous
TT: Tuberculoid
BI: Bacterial index
MB: Multibacillary
BCG: Bacillus Calmette-Guérin
BT: Borderline – tuberculoid
BB: Mid – borderline
BL: Borderline - lepromatous
PB: Paucibacillary
HHC: Household contacts
ELISA: Enzyme-linked immunosorbent assay
OPD: Ortho-phenylenediamine
EI: ELISA index
OD: Optical density
H: Kruskal-Wallis one-way coefficient
rho: Spearman coefficient
GPop: General population
K: Kappa coefficient
